# Comparison of the Proteomes of Three Yeast Wild Type Strains: CEN.PK2, FY1679 and W303

**DOI:** 10.1002/cfg.94

**Published:** 2001-08

**Authors:** Adelina Rogowska-Wrzesinska, Peter Mose Larsen, Anders Blomberg, Angelika Görg, Peter Roepstorff, Joakim Norbeck, Stephen John Fey

**Affiliations:** 1 Centre for Proteome Analysis in Life Sciences University of Southern Denmark International Science Park Odense Forskerparken 10B Odense M 5230 Denmark; 2 Deptartment of Cell and Molecular Biology – Microbiology Lundbergs Laboratory Göteborg University Medicinaregatan 9C Göteborg 405 30 Sweden; 3 Technical University of Munich Institute of Food Technology and Analytical Chemistry Proteomics Group Freising-Weihenstephan D-85350 Germany; 4 Department of Biochemistry and Molecular Biology University of Southern Denmark Odense University, Campusvej 55 Odense M 5230 Denmark; 5 Institut fuer Biochemie und Molekulare Zellbiologie Universität Wien Vienna Dr-Bohrgasse 9 A-1030 Austria

## Abstract

Yeast deletion strains created during gene function analysis projects very often show drastic phenotypic differences depending on the genetic background used. These results indicate the existence of important molecular differences between the CEN.PK2, FY1679 and W303 wild type strains. To characterise these differences we have compared the protein expression levels between CEN.PK2, FY1679 and W303 strains using twodimensional gel electrophoresis and identified selected proteins by mass spectrometric analysis. We have found that FY1679 and W303 strains are more similar to each other than to the CEN.PK2 strain. This study identifies 62 proteins that are differentially expressed between the strains and provides a valuable source of data for the interpretation of yeast mutant phenotypes observed in CEN.PK2, FY1679 and W303 strains.


**Comparative and Functional Genomics**
*Comp Funct Genom* 2001; **2**: 207–225.
**DOI:** 10.1002/cfg.94

**Research Article**

### Comparison of the proteomes of three yeast wild type strains: CEN.PK2, FY1679 and W303

Adelina Rogowska-Wrzesinska[1]*, Peter Mose Larsen[1], Anders Blomberg[2], Angelika Görg[3], Peter Roepstorff[4], Joakim Norbeck[5] and Stephen John Fey[1]

[1] *Centre for Proteome Analysis in Life Sciences, University of Southern Denmark, International Science Park Odense, Forskerparken 10B, 5230 Odense M, Denmark*
[2] *Deptartment of Cell and Molecular Biology – Microbiology Lundbergs Laboratory, Göteborg University, Medicinaregatan 9C 405 30 Göteborg, Sweden*
[3] *Technical University of Munich, Institute of Food Technology and Analytical Chemistry, Proteomics Group, D-85350 Freising-Weihenstephan, Germany*
[4] *Department of Biochemistry and Molecular Biology, University of Southern Denmark, Odense University, Campusvej 55, 5230 Odense M, Denmark*
[5] *Institut fuer Biochemie und Molekulare Zellbiologie, Universität Wien, Dr-Bohrgasse 9 A-1030 Vienna, Austria*

**Correspondence to: A. Rogowska-Wrzesinska, Centre for Proteome Analysis in Life Sciences, University of Southern Denmark, International Science Park Odense, Forskerparken 10B, 5230 Odense M, Denmark. E-mail: ar@cpa.spo.dk*

Received: 1 May 2001
Accepted: 26 June 2001

### Abstract

**Yeast deletion strains created during gene function analysis projects very often show drastic phenotypic differences depending on the genetic background used. These results indicate the existence of important molecular differences between the CEN.PK2, FY1679 and W303 wild type strains. To characterise these differences we have compared the protein expression levels between CEN.PK2, FY1679 and W303 strains using two-dimensional gel electrophoresis and identified selected proteins by mass spectrometric analysis. We have found that FY1679 and W303 strains are more similar to each other than to the CEN.PK2 strain. This study identifies 62 proteins that are differentially expressed between the strains and provides a valuable source of data for the interpretation of yeast mutant phenotypes observed in CEN.PK2, FY1679 and W303 strains.** **Copyright © 2001 John Wiley & Sons, Ltd.**

**Keywords:** ***Saccharomyces cerevisiae*; CEN.PK2; FY1679; W303; protein expression; proteomics; two-dimensional gel electrophoresis**

### Introduction

The availability of the complete genomic sequence of the yeast *Saccharomyces cerevisiae* opened the possibility to systematically study gene function and gave a unique insight into the molecular basis of function and growth of a single *eukariotic* cell. An international effort was initiated with the aim of creating a fundamental tool for such functional analysis, a collection of yeast genomic disruptants and plasmids. By August 2000, 22 472 yeast deletion strains covering 5867 different genes plus 1309 plasmids had been collected in the EUROSCARF collection generated by the German functional analysis project, Eurofan I and Eurofan II projects (**http://www.rz.uni-frankfurt.de/FB/fb16/mikro/euroscarf/index.html**). During the functional analysis projects, the deletions have been carried out in four different genetic backgrounds: FY1679 (isogenic to S288C whose DNA was sequenced), CEN.PK2 (generated in the German functional analysis project), W303 used in EUROFAN I and in the BY-series of strains (also isogenic to S288C strain).

Since yeast deletion strains can show drastic phenotypic differences depending on their genetic background, for example the null mutants of *SSH1* gene exhibit slow growth at 30°C and 37°C in the FY1679 and W303 strains, but have normal growth in the CEN.PK2 strain (Duenas *et al.*, 1999), it is important to be aware of strain-related differences (for further examples see Table 1).

The surprising fact is that although many

Copyright © 2001 John Wiley & Sons, Ltd.

Table 1. Examples of genes exhibiting strain-dependent mutant phenotypes. Gene name and function are cited from the YPD protein database (Costanzo *et al.*, 2000)

| Gene name | Mutant phenotype in CEN.PK2 background | Mutant phenotype in FY1679 background | Mutant phenotype in W303 background | Reference | Protein function |
|---|---|---|---|---|---|
| SUB2 | Slow growth | Lethal | Not determined | Lopez *et al.*, 1998 | RNA splicing, ATP dependent RNA helicase |
| PKH2 | Lethal | No defect | Not determined | Bilsland *et al.*, 1998 | Ser/thr protein kinase |
| ASM4 | No defect | Lethal | Not determined | Lopez *et al.*, 1998 | Component of karyopherin docking complex of the nuclear pore |
| ARP7 | Not determined | Lethal in S288C | Slow growth | Cairns *et al.*, 1998 | Swi-snf global transcription activator complex |
| ARP9 | Not determined | Lethal in S288C | Slow growth | Cairns *et al.*, 1998 | Swi-snf global transcription activator complex |
| HSL7 | No defect | No defect | Abnormalities in budding | Kucharczyk *et al.*, 1999 | Negative regulator of SWE1p, mitosis and cell cycle |
| SSH1 | No defect | Slow growth | Slow growth | Duenas *et al.*, 1999 | Protein translocation, ssh1-sss1-sbh2 complex interacting with ribosomes |
| DCP1 | Slow growth | Lethal | Not determined | Hajji *et al.*, 1999 | mRNA decapping enzyme involved in mRNA turnover |
| NHP6A NHP6B Double mutant | Not determined | Lethal in S288C | Grows on galactose | Yu *et al.*, 2000 | RNA Polymerase II transcription |
| GAL4 | Not determined | Ser699 phosphorylation required for GAL induction | Ser699 phosphorylation not required for GAL induction | Rohde *et al.*, 2000 | Transcription factor, phosphorylation correlates with activity |
| ARP5 | Not determined | Severe growth defect | Lethal | Grava *et al.*, 2000 | Actin-related protein |

laboratories all around the world use these yeast strains, very little is known about how they compare to each other.

### Genealogy and characteristics of CEN.PK2, FY1679 and W303 yeast strains

FY1679 strain was constructed by crossing FY23 and FY73 haploid strains ($23 \times 73 = 1679$), (Thierry *et al.*, 1990). Strains FY73 (*MATα ura3-52, his3-Δ200, GAL2*) and FY23 (*MATa ura3-52, trp1-Δ63, leu2-Δ1 GAL2*) are isogenic derivatives of strain S288C constructed by gene replacement (Winston *et al.*, 1995). FY1679 was used as a source of DNA for a library that has been used for the European Union Yeast Genome Sequencing Programme.

The origin of S288C strain has been described by Mortimer and Johnston (1986). They have determined that the principal progenitor strain of S288C was the strain EM93, which contributes approximately 88% of the gene pool in S288C. EM93 was originally isolated by E. Mark from rotting figs near Merced, California (Lindegarden, 1949). There were other strains that contributed to the genetic pool of S288C: EM126 (*Saccharomyces carlsbergiensis*); NRRL-210 isolated from rotting bananas from Costa Rica in 1942 (C.Kurtzman); FLD-commercial baking yeast and LK (Lindegarden, 1949) and yeast foam (Ephrussi, Hottingner, Tavlitzki, 1949) – both baking yeast (Figure 1). For details on the genealogy of S288C strain see (Mortimer and Johnston, 1986). In fact S288C was constructed by RK Mortimer by genetic crosses as a parental strain for biochemical mutants (Mortimer and Johnston, 1986). Requirements for the strain were that it must be non-clumpy – dispersing into single cells in liquid culture and have a minimal number of nutritional requirements. S288C requires only biotin, nitrogen source, glucose, salts and trace elements.

Several studies have characterised different genetic properties of S288C strain. Here are just some examples. The S288C strain is unable to grow pseudohyphally because it carries a nonsense mutation in *FLO8* gene that is necessary for

Copyright © 2001 John Wiley & Sons, Ltd. 

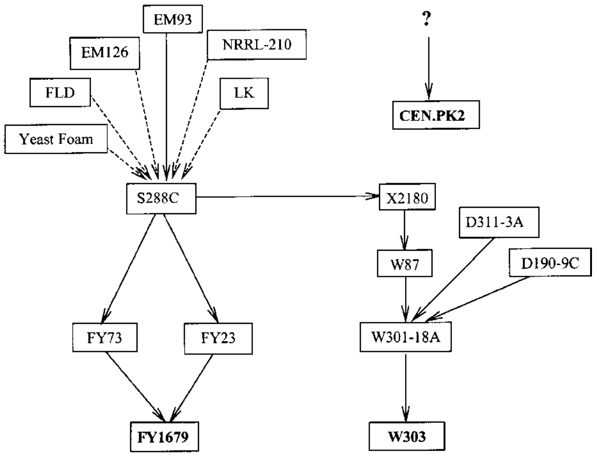

**Figure 1.** Genealogy of basic laboratory yeast strains: CEN.PKC, FY1679 and W303. The dotted arrows indicate that the contribution of these strains to the genealogy of the strain S288C is not fully determined filamentous growth (Liu *et al.*, 1996 and Kron *et al.*, 1997). The *HAP1* gene coding for a haem-dependent transcription factor in S288C and strains derived from it carries a Ty1 insertion which results in replacement of 13 amino acids (Gaisne *et al.*, 1999). The strain S288C has only one copy of the *NHA1* gene (putative Na+/H+ antiporter), whereas W303 appears to have more than one copy (Prior *et al.*, 1996). Two genes encoding killer toxins, *KHR1* and *KHS1* are modified in S288C. The *KHR1* gene is absent in strain S288C and *KHS1* is interrupted by multiple frameshifts (Goto *et al.*, 1991). Strain S288C has a null mutation in the *KSS1* gene, which is involved in the filamentous and invasive growth pathway (Elion *et al.*, 1991). Most laboratory strains, including S288C have substitutions in *AQY1* gene and are interrupted in *AQY2*, which cause reduced water transport activity (Bonhivers *et al.*, 1998). *MEL1*, *MEL2*, *MEL5* and *MEL6*, which are involved in melibiose utilisation are not found in S288C (Lieberman *et al.*, 1991). FY1679 has a poor sporulation frequency compared to CEN.PK2 and W303 strains (Entian and Kotter, 1998).

The diploid strain W303 was constructed by transforming haploid strain W301-18A with a plasmid containing the HO gene and screening for diploids after loss of the plasmid (Thomas and Rothstein, 1989; Wallis *et al.*, 1989). The diploid strain was dissected to obtain the isogenic *MATa* and *MATα* strains, W303-1A and W303-1B. Unfortunately, the genealogy of strain W301-18A has not been described before (Rothstein, 1983). Here we can conclude that W303 and S288C are very similar (as stated by Rodney Rothstein, personal communication). In fact, W303-18A was constructed by many crosses of W87 strains (Rothstein, 1977; Rothstein *et al.*, 1977), which are mainly, but not exclusively, descendants of the strain X2180, itself derived from S288C by self – diploidisation (Mortimer and Johnston, 1986). Part of the genetic background of W301-18A was also obtained from the D311-3A strain constructed by Fred Sherman (Rothstein and Sherman 1980a,b). Additionally, one of the grandparents of W301-18A was the D190-9C strain that was obtained from Jack Szostak and about which is very little known (personal communication, Rodney Rothstein) (Figure 1).

The genotype of the W303 strain is *MATa/MATα* (*leu2-3,112 trp1-1 can1-100 ura3-1 ade2-1 his3-11,15*) [phi+]. The [phi+] element is a non-Mendelian segregating trait that affects the efficiency of suppression of amber stop codon. Unlike the related element [psi+], this element does not affect suppression of ochre stop codons. *Ade2-1* and *can1-100* are ochre suppressible, *trp1-1* is amber suppressible and *ura3-1* reverts at very low frequency ($2 \times 10^{-9}$). Both *leu2-3,112* and *his3-11,15* do not revert at any measurable frequency (personal communication, Rodney Rothstein). It has a very weak allele of *RAD5* gene, which was discovered by Hannah Klein, and unlike S288C, W303 is wild type for *KSS1* (personal communication, Rodney Rothstein).

The strain CEN.PK2 was developed specially for functional analysis by K-D Entian *et al.* (1999) but its origin and progenitor strain have not been published (Entian and Kotter, 1998). The sporulation efficiency of CEN.PK2 is as good as that of the W303 strain, and it has a faster growth rate, with doubling times of about 80 min for haploid strains (Entian and Kotter, 1998).

### Background dependent yeast deletant phenotypes

The large number of examples of genetic background-dependent mutant phenotypes and differences between wild-type strains shows the importance of understanding the molecular characteristics of these wild-type strains. Although, various basic phenotypic analyses of specific yeast deletants revealed significant differences between the background strains (Table 1), only one study

Copyright © 2001 John Wiley & Sons, Ltd. 

has been carried out to characterise some of these differences. Günter Daum and co workers (Daum *et al.*, 1999) have shown that CEN.PK2, FY1679-28C and W303 strains exhibit different levels of sterols and triacylglycerols. Thus there is an urgent need to thoroughly characterise these three laboratory wild type strains.

### Proteome analysis by two dimensional gel electrophoresis (2DGE) and mass spectrometry

The recent developments of 2DGE and associated tools, such as mass spectrometry and software programs dedicated to image analysis, offer novel opportunities for studying the genetic background of different yeast strains (Joubert *et al.*, 2000). Two-dimensional electrophoresis separates proteins in terms of their isoelectric point and molecular mass. When applied to yeast whole cell extracts it can resolve several thousand proteins (Fey *et al.*, 1997; Nawrocki *et al.*, 1998). Hence 2DGE provides an opportunity to analyse a global picture of a given yeast strain under given environmental conditions. Several protein maps of *Saccharomyces cerevisiae* strains have been made (Boucherie *et al.*, 1996; Shevchenko *et al.*, 1996; Nawrocki *et al.*, 1998; Garrels *et al.*, 1997; Norbeck and Blomberg, 1997; Perrot *et al.*, 1999) and several hundreds of proteins have been identified on these maps, proving that 2DGE is a very powerful tool for analysing the yeast proteome.

In this study, we have compared the protein map of three wild-type strains, CEN.PK2, FY1679-28C and W303, which are widely used for functional analysis of yeast genes. We demonstrate that there are 64 pronounced differences in the expression patterns between these strains, some of which have previously been found at the transcriptome level. The results show that FY1679 and W303 strains are more closely related to each other than to the CEN.PK2 strain.

### Materials and methods

### Materials

Immobilised pH-gradient strips covering pH 4–7, 6–9, 4.5–5.5 and 5.5–6.7, pharmalytes 3–10 (Code No. 17-0456-01) and IPG buffer 6–11 (Code No. 17-6001-78), [$^{35}$S]-methionine were from Amersham Pharmacia Biotech. Acrylamide used for 2$^{nd}$ dimension gels was from Genomic Solutions, Bis N,N′-Methylene-bis-acrylamide from Bio Rad. Chemicals used for lysis buffer and equilibration buffer were as follows: urea (ICN Biomedicals), thiourea (Fluka), chaps (Sigma), DTT (Sigma), SDS (Serva). Yeast nitrogen base w/o amino acids was obtained from Difco, and all amino acids were from Sigma.

### Yeast culture and media

The yeast *Saccharomyces cerevisiae* diploid strains CEN.PK2 (*Mat a/α, ura3-52, leu2-3,112, trp1-289, his3-1*), FY1679 (*Mat a/α, ura3-52/ ura3-52, leu2-1/ +, trp1-63/+, his3-200/+*) and W303 (*Mat a/α, ura3-1, leu2-3,112, trp1-1, his3-11,15, ade2-1, can1-100*) were obtained from EUROSCARF collection. The diploid strains were sporulated to produce haploid strains of mating type α with all the possible phenotypic markers to make them as similar as possible. The haploid strains selected for this study were called CEN.PK2-1B (*Matα, ura3-52, leu2-3,112, trp1-289, his3-1*), FY1679-1D (*Matα, ura3-52, trp1-63, leu2-1, his3-200*) and W303-1B (*Matα, ura3-1, leu2-3,112, trp1-1, his3-11,15, ade2-1, can1-100*) in this manuscript. Initially, the strains were grown on agar plates (1% yeast extract, 1% bactopeptone, 2% bactoagar, 2% glucose) and three single cell colonies of each strain were isolated and named A, B and C. Auxotrophic tests were performed as a quick test to check that the strains were carrying the right markers. For all experiments cells were grown in YNB-Glucose medium (0,67% YNB w/o amino acids, 1.6% w/v sodium hydroxide-succinate buffer pH 5.8, 2% glucose) supplemented with 0.2 mg/ml of uracil, tryptophane, histindine, adenine and 0.1 mg/ml of leucine.

### Labelling of proteins and protein extraction

The cell culture was incubated on a rotary shaker shaking at 200 rpm at 28°C. Growth was monitored by following the optical density (OD) of the culture measured as light scattering at 600 nm wavelength (GENESYS 2, Spectronic Instruments). As the culture reached the OD = 0.35 (approx. $5 \times 10^6$ cells per ml), 3 ml of culture was transferred to a 10ml flask and labelled for 30 min by adding 100 μCi of [$^{35}$S]-methionine. The cells were centrifuged, washed with 1.5 ml of distilled water and the yeast pellet was resuspended in 120 μl of lysis buffer (7 M urea, 2 M thiourea, 2% CHAPS, 0.4% DTT, 1% v/v pharmalytes 3–10). Cells were sonicated on a

Copyright © 2001 John Wiley & Sons, Ltd. 

melting-ice ethanol bath, at an amplitude of 6 microns, 2 times for 5s with 30s break, as described before (Nawrocki *et al.*, 1998) and left shaking at room temperature overnight. Unlabelled samples (for preparative gels) were prepared in the same way with the exception that 50 ml of culture were used and [$^{35}$S]-methionine was not added.

### Protein and CPM determination

The protein concentration in the samples was determined using the Bradford method (Bradford, 1976), which was adapted for use with lysis buffer as previously described (Fey *et al.*, 1997). Determination of isotope incorporation into proteins was performed using trichloroacetic acid precipitation as described before (Fey *et al.*, 1997).

### 2D gel electrophoresis

1$^{st}$ dimension gel electrophoresis was performed on 18 cm long IPG4–7 and IPG6–9 gradient gels. The rehydration buffer for IPG4–7 strips was identical to the lysis buffer used for sample preparation and the sample was applied by in-gel rehydration. IPG6–9 gels were rehydrated in similar buffer except that 0.5% v/v pharmalytes 3–10 and 0.5% v/v pharmalytes 6–11 were added and the sample was applied by cup-loading. $2 \times 10^6$ CPM were loaded on each gel.

Focusing was performed on Multiphor II at 20°C using a voltage/time profile linearly increasing from 0 V to 600 V for 2 : 15 h, from 600 V to 3500 V for 1 h and 3500 V for 13 : 30 h for IPG4–7 or for 3 h for IPG6–9. After focusing, strips were equilibrated twice for 15 min in equilibration buffer (6 M urea, 2% SDS, 30% Glycerol, 50 mM Tris-HCl pH 8.8, 1% DTT). For convenience, the gels were kept frozen at −80°C between the equilibration steps.

SDS PAGE second dimension gel electrophoresis was performed using a vertical Investigator™ 2-D Electrophoresis System (Millipore) and laboratory-made 12.5% (w/v) acrylamide gels (acrylamide: N, N′-ethylene-bis-acrylamide ratio 200 : 1). The gels were run overnight at 20°C at a constant current setting. The running buffer was recirculated using an aquarium pump (flow rate nominally 4 l per min).

### Pattern visualisation and computer analysis

After the second dimension separation, gels were dried directly on 3 mm Whatmann paper, exposed to Phosphoimager plates (AGFA) for 120 hours, and read in an AGFA ADC70 reader. The 2D gel patterns obtained have been compared to our existing database (Nawrocki *et al.*, 1998) and other yeast 2D databases (Norbeck and Blomberg, 1997; Perrot *et al.*, 1999; Hoogland *et al.*, 2000) and verified in relation to known identified proteins.

Three images of each strain were matched and compared with images of other strains using Image Master (Amersham Pharmacia Biotech) computer program. Protein expression is measured as the sum of all the pixel grey values within the spot boundary (integrated optical density, IOD). This is then given as a percentage of the total of all the spots (%IOD). Boundaries, for spots that are present in one strain and missing in another have been added into the correct position in the latter images so that the background values can be used for statistical purposes. On each gel image we analysed the same number of spots (1222 on IPG4–7 gels and 389 on IPG6–9 gels).

The average %IOD and standard deviation was calculated from the expression data from the 3 corresponding images for each strain. These were then compared for each spot between each pair of strains using the Student's *t*-test, to reveal proteins whose expression was statistically different ($p > 99\%$).

In order to estimate the reproducibility of the 2D gel system, the average percentage standard deviation for all the spots of each strain and each gel system used was calculated. This ranged from 35% to 44% for the IPG4–7 gels and from 28% to 35% for IPG6–9 gels. Thus the high reproducibility of the protein patterns allowed a reliable selection of spots differing by a 40% change in spot intensity (factor 1.4 or more and 0.71 or less). These two criteria were used together to select differences that were significant at the level of 99% and which differed by at least factor 1.4 or less than factor 0.71 between at least two of the analysed strains. Each selected difference was visually evaluated to make sure that the spot detection and matching were correct.

### Preparative and zoom gels

For protein identification by mass spectrometry, preparative IPG4–7 and IPG6–9 gels were loaded with 200µg of cold proteins in addition to the [$^{35}$S]-methionine labelled proteins and were run using the same gradients and procedures. Three preparative gels were made for each strain. In addition to the standard gradients, two one-pH unit zoom gradient gels, IPG4.5–5.5 and IPG5.5–6.7, were used for

Copyright © 2001 John Wiley & Sons, Ltd. 

protein identification. Zoom gels were loaded with 300 μg of cold protein and $4 \times 10^6$ CPM of [$^{35}$S]-methionine labelled proteins. The 1$^{st}$ dimension was performed using the same procedures as for IPG4–7 and 6–9 gradients with the following running profile: voltage/time profile linearly increasing from 0 V to 600 V for 2 : 15 h, from 600 V to 3500 V for 1 h and 3500 V for 21 : 45 h. After the 2$^{nd}$ dimension, preparative and zoom gels were dried and exposed to X-ray film for 10 days.

### Mass spectrometric protein identification

Proteins of interest were manually excised from preparative and zoom gels, and after in-gel digestion they were analysed by MALDI mass spectrometry (Jensen *et al.*, 1998). The mass spectra obtained were internally calibrated using trypsin autodigestion peptides and then used to search the NCBI database using the MASCOT (**http://www.matrixscience.com**), MS-Fit (**http://prospector.ucsf.edu**) and ProFound (**http://www.proteometrics.com**) search programs. Database searches were performed using the following parameters with minor modifications needed for each program: all species, no restrictions for molecular weight and protein pI were used, trypsin digest, one missed cleavage allowed, cysteines modified by acrylamide allowed, oxidation of methionines possible, mass tolerance between 0.1–0.5 Da. An identification was considered positive when at least 5 peptides were matching with no sequence overlap.

### Results and discussion

This study was performed in triplicate: each strain was grown from three independent cultures, each derived from a single cell colony. These were grown, labelled and after protein extraction independently analysed by 2DGE. The replicates were used as independent experiments during the statistical analysis of the spot intensity data. Consequently, the proteome of each strain was analysed and quantitated using three IPG4–7 and three IPG6–9 gels (Figure 2A, B and C). The overlapping region between the gels was analysed on both gradients.

During the computer assisted image analysis we have detected and matched 1222 spots on IPG4–7 and 389 spots on IPG6–9 gradient gels. Comparison and statistical analysis of the nine IPG4–7 and 9 IPG6–9 [$^{35}$S]-methionine labelled protein 2DGE patterns from analysed yeast strains revealed: 73 protein spots significantly changed between CEN.PK2-1B and FY1679-1D; 67 spots significantly changed between CEN.PK2-1B and W303-1B; and only 39 spots changed between FY1679-1D and W303-1B. In total, 122 protein spots were significantly changed between at least two of the three studied strains. The presented data and the genealogy of the strains both indicate that FY1679-1D and W303-1B are more similar to each other than to the CEN.PK2-1B strain.

The selected protein spots have been divided into four categories (A, B, C and ABC) depending on the relative intensity of selected protein expression (Table 2). These correspond to proteins that are either up (+) or down (−) regulated in respectively CEN.PK2-1B (45 spots), FY1679-1D (25 spots) or W303-1B (17 spots) strains compared to the other two strains. The last category (ABC) contains 35 spots that show different expression levels in all three strains. The last group has been divided into subgroups where the order of the letters indicates the order of decreasing abundance of the protein. The large group of proteins that exhibit changes in all the strains indicates that the protein expression of some genes is not tightly regulated between the strains.

### Mass spectrometric protein identification

All protein spots that exhibited significant intensity differences (122) between at least two of the analysed strains were subjected to MALDI mass spectrometric analysis irrespective of their abundance. In the first attempt spots were cut out from preparative IPG4–7 and IPG6–9 gels. Each spot was cut from the gel of the strain where the protein was most abundant.

For some of the spots, with relatively weak spot intensity, we could obtain only a few peptide mass peaks, which were not sufficient to unambiguously identify the protein. There were also other spots, which contained more than one protein. To identify some of these spots we have run narrow range IPG4.5–5.5 and IPG5.5–6.7 preparative gels and cut the remaining spots from them.

In total, in this work we have identified proteins in 101 of the spots (14 of which contained more than one protein), which represents an 82.7% rate of identification. The spots selected for cutting can be divided into three intensity groups. Spots of low intensity (<0.066 of %IOD) comprising 48 spots; spots of medium intensity (between 0.066 and 0.198 %IOD) comprising 50 spots and high

Copyright © 2001 John Wiley & Sons, Ltd. 

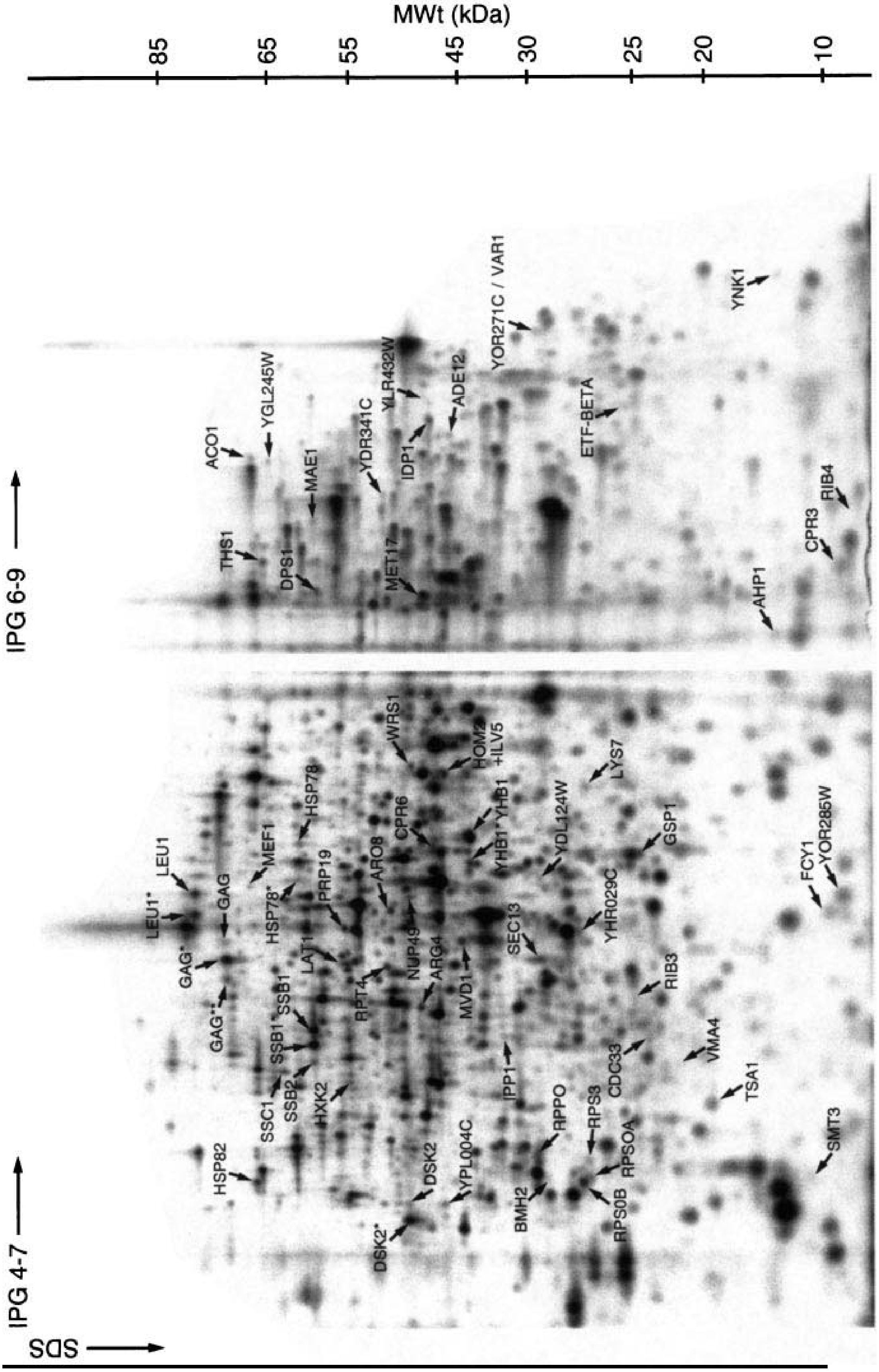

MWt (kDa)
85
65
55
45
30
25
20
10
IPG 6-9
IPG 4-7
SDS
ACO1
YGL245W
THS1
MAE1
DPS1
YDR341C
YLR432W
IDP1
ADE12
MET17
YOR271C / VAR1
ETF-BETA
YNK1
AHP1
CPR3
RIB4
LEU1*
LEU1
GAG*
GAG
GAG*
MEF1
HSP78*
HSP78
PRP19
LAT1
SSB1*
SSB1
SSC1
SSB2
HXK2
HSP82
RPT4
ARO8
NUP49
ARG4
CPR6
WRS1
HOM2
+ILV5
YHB1*
YHB1
MVD1
SEC13
YDL124W
LYS7
YHR029C
GSP1
RIB3
IPP1
CDC33
VMA4
TSA1
RPPO
RPS3
RPSOA
RPS0B
BMH2
YPL004C
DSK2
DSK2*
FCY1
YOR285W
SMT3

Figure 2a

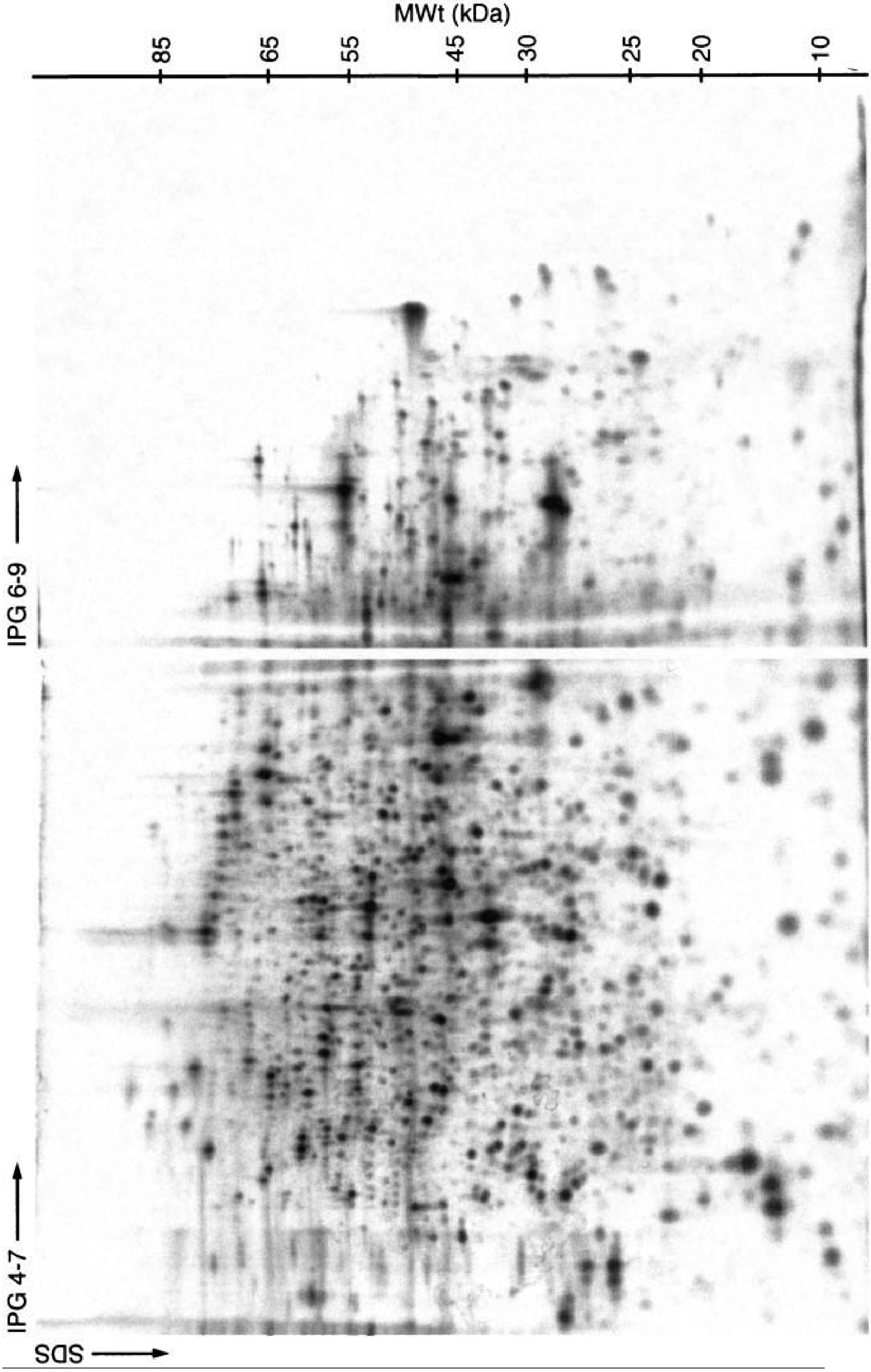

Figure 2b

Copyright © 2001 John Wiley & Sons, Ltd.

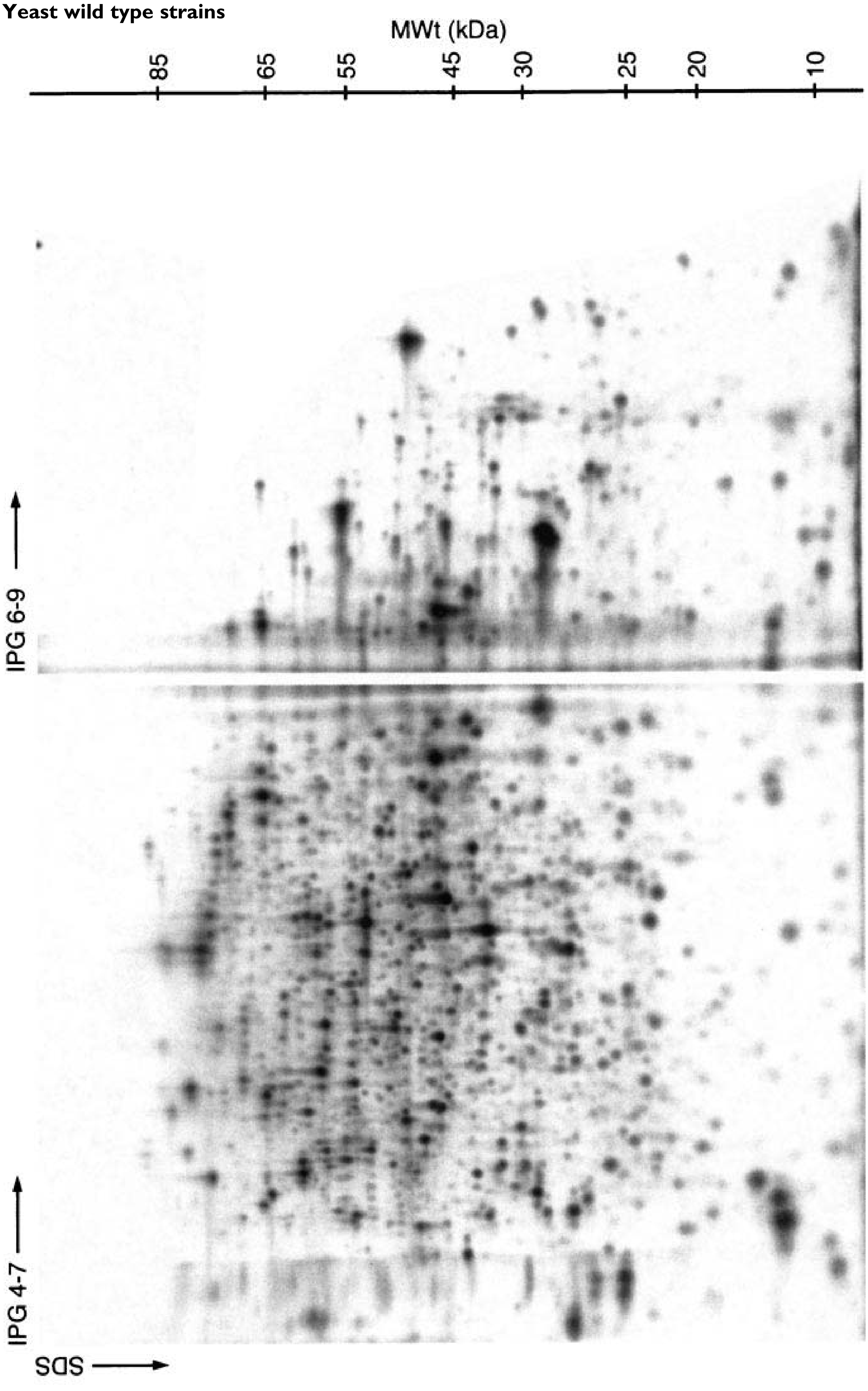

Figure 2c

Copyright © 2001 John Wiley & Sons, Ltd.

intensity spots ($>0.198$ %IOD) comprising 24 spots. The identification rate in these groups was 73.3% (33), 90% (55) and 95.8% (23 spots) respectively. These results clearly show that most of the unidentified spots belong to the group of weak spots. To confirm this, the radioactively labelled gels were compared with silver stained gels of the same strains loaded with 150 μg of protein. This comparison has shown that the spots from group of low intensity spots were in most cases very small or hardly visible on silver stained gels, confirming the high sensitivity of the mass spectrometric methods used.

### Truncated proteins

Some of the spots were identified as a mixture of two or three protein components. Usually one of the two components was identified by a higher number of matching peptides and a better sequence coverage. Sometimes the other protein was a breakdown product of a larger protein with peptide masses covering only a fragment of the protein sequence. Some other protein spots contained only a fragment of a larger protein. Whether these protein fragments are generated by specific protein degradation involved in the control of protein turnover or some post translation processing, or are induced during sample preparation remains to be determined. It is, however, very unlikely that the observed protein fragments are generated randomly and introduced by sample preparation and handling, because we observed reproducible, statistically significant differences between the intensities in the strains we analysed.

### Proteome differences between the CEN.PK2-1B, FY1679-1D and W303-1B strains

Figure 2A and Table 2 show all of the spots exhibiting changes in expression between the CEN.PK2-1B, FY1679-1D and W303-1B strains that were unambiguously identified, and showed no evidence of protein truncation. These 66 spots contained 62 different proteins, four spots contained 2 proteins (Hom2p and Ilv5p; Rpn12p and Rps0ap; Ade12p and Gnd1p; Var1p and Yor271cp), 5 proteins were found to be present in two spots (Dsk2p, Leu1p, Hsp78p, Yhb1p and Ssb1p) and 1 protein (Gagp) was identified as being present in 3 spots. The identified proteins can be divided into 7 groups according to their cellular location: cytoplasmic (17), mitochondrial (13), nuclear (11), ribosomal (6), unknown (15), lysosomal (1); vacuolar (1); and one virus protein. These proteins can also be divided into functionally related groups, as indicated in Table 2.

There is unfortunately no space in this article to discuss all the protein groups and the possible implications for the phenotypes of the analysed strains and of deletion mutants made in these background strains. Therefore we have selected only some of the proteins for detailed analysis and discussion.

### Major coat protein from *Saccharomyces cerevisiae* virus ScV-L-A

In this study 3 protein spots that lay in a row very close to each other were selected. All three spots were present in the CEN.PK2-1B strain and were not visible in gels from the FY1679-1D and W303-1B strains (Figure 3).

MALDI mass spectrometric analysis revealed that all three spots contain Gagp – the major coat protein from *Saccharomyces cerevisiae* virus L-A (ScV-L-A) indicating that the CEN.PK2-1B strain contains active ScV-L-A virus.

It has been reported previously that N-terminal acetylation of the Gagp is necessary for viral assembly and that the yeast Mak3p N-acetyltransferase is responsible for that modification (Tercero *et al.*, 1993). In our study the presence of three spots representing the Gagp protein indicates a post-translational modification of this protein. To confirm this we re-examined the MALDI mass spectrometry results. Unfortunately the N-terminal peptide resulting from digestion of Gagp with trypsin consists of three amino acids and has a theoretical mass of only 418.2 Da. In this range, the background noise created by matrix ions in the MALDI mass spectrum is too high to distinguish protein peaks. Therefore we were not able to

**Figure 2.** 2DGE pattern of [$^{35}$S]-methionine labelled proteins from yeast *Saccharomyces cerevisiae*: a) CEN.PK2-1B strain total cell lysate. b) FY1679-1B strain total cell lysate. c) W303-1B strain total cell lysate. Proteins were separated using IPG4–7 and IPG6–9 gradient in the 1$^{st}$ dimension (horizontal) and by SDS-PAGE electrophoresis in 12.5% polyacrylamide gels in the 2$^{nd}$ dimension (vertical). Names indicate all proteins listed in Table 2. Star (*) following protein name indicates observed protein isoform

Copyright © 2001 John Wiley & Sons, Ltd.

Table 2. Functional classification of proteins differently expressed between yeast wild type strains CEN.PKC-1B, FY1679-1D and W303-1B. The table lists all the proteins that have been identified by mass spectrometry, and neither their spot position, nor the mass spectrometry peptide coverage map indicated protein truncation. The proteins were identified in spots that exhibited intensity differences at significance level of 99% and differed by at least factor 1.4 or less than factor 0.71 between at least two of the analysed strains. The expression groups divide proteins into categories based on the spots percentage integrated optical density (%IOD). The 4 categories (A, B, C and ABC) depend on the relative intensity of selected protein expression. These correspond to proteins that are either up (+) or down (−) regulated in CEN.PK2-1B, FY1679-1D or W303-1B strains respectively, compared to the other two strains. The last category (ABC) contains spots that show different expression levels in all 3 strains. The last group has been divided into subgroups where the order of the letters indicates the order of decreasing abundance of the protein. Cellular localisation, protein name and biochemical function and cellular role are cited from the YPD protein database (Costanzo *et al*., 2001). Protein names marked with stars (*) indicate protein isoforms shown in Figure 2

| **Protein** | **Cellular localisation** | **Protein name and biochemical function** | **Cellular role** | **Expression group** | **Average %IOD in CEN.PK2-1B** | **Average %IOD in FY1679-1D** | **Average %IOD in W303-1B** |
|---|---|---|---|---|---|---|---|
| ScV-L-A VIRUS | | | | | | | |
| Gagp | Virus | Major coat protein ScV-L-A virus | Virus | A+ | 0.067 | 0.016 | 0.020 |
| Gagp* | Virus | Major coat protein ScV-L-A virus | Virus | A+ | 0.183 | 0.007 | 0.012 |
| Gagp** | Virus | Major coat protein ScV-L-A virus | Virus | A+ | 0.084 | 0.025 | 0.004 |
| NUCLEAR CYTOPLASMIC TRANSPORT | | | | | | | |
| Gsp1p | Nuclear | GTP-binding protein of ras superfamily, nuclear transport, export of 60S ribosomal subunits | Nuclear-cytoplasmic transport | A+ | 0.104 | 0.073 | 0.068 |
| Nup49p | Nuclear | Nuclear pore protein, nuclear import/export | Nuclear-cytoplasmic transport | C+ | 0.007 | 0.005 | 0.020 |
| SMALL MOLECULE TRANSPORT | | | | | | | |
| Sec13p | Cytoplasimc | Component of COPII coat of vesicles involved in endoplasmic reticulum to Golgi transport | Small molecule transport, vesicular transport | A+ | 0.094 | 0.007 | 0.015 |
| Vma4p | Lysosome/ vacuole | Vacuolar H(+)-ATPase, hydrophilic subunit; hydrolase and transporter | Small molecule transport | B+ | 0.006 | 0.212 | 0.001 |
| Yor271cp | Mitochondrial | Member of the mitochondrial tricarboxylate carrier family of membrane transporters | Small molecule transport | A+ | 0.117 | 0.053 | 0.049 |
| CELL CYCLE, DNA REPAIR, MITOSIS AND SIGNAL TRANSDUCTION | | | | | | | |
| Cdc33p | Nuclear | Translation initiation factor eIF4E, mRNA cap binding protein found in association with Caf20p | Cell cycle control, protein synthesis | C+ | 0.046 | 0.044 | 0.062 |
| Prp19p | Nuclear | Non-snRNP spliceosome component, also involved in mitotic recombination and gene conversion | DNA repair, RNA splicing, RNA processing | A+ | 0.051 | 0.019 | 0.016 |

Copyright © 2001 John Wiley & Sons, Ltd.

Table 2. Continued

| Protein | Cellular localisation | Protein name and biochemical function | Cellular role | Expression group | Average %IOD in CEN.PK2-1B | Average %IOD in FY1679-1D | Average %IOD in W303-1B |
|---|---|---|---|---|---|---|---|
| Dsk2p | Nuclear | Protein required with Rad23p for duplication of the spindle pole body; has similarity to ubiquitin | Mitosis | A− | 0.066 | 0.152 | 0.157 |
| Dsk2p* | Nuclear | Protein required with Rad23p for duplication of the spindle pole body; has similarity to ubiquitin | Mitosis | A+ | 0.191 | 0.082 | 0.074 |
| Bmh2p | Unknown | Homolog of 14-3-3 protein, involved in signal transduction and differentiation | Signal transduction, differentiation | B+ | 0.023 | 0.054 | 0.030 |
| ENERGY GENERATION IN MITOCHONDRIA | | | | | | | |
| Mae1p | Mitochondrial | Mitochondrial malate dehydrogenase | Energy generation, carbohydrate metabolism | A+ | 0.122 | 0.045 | 0.016 |
| Aco1p | Mitochondrial | Aconitate hydratase (aconitase) converts citrate to cis-aconitate | Energy generation, carbohydrate metabolism, Amino acid metabolism | C− | 0.086 | 0.082 | 0.020 |
| Idp1p | Mitochondrial | Isocitrate dehydrogenase (NADP+) | Energy generation | ABC | 0.447 | 0.380 | 0.225 |
| Lat1p | Mitochondrial | Dihydrolipoamide S-acetyltransferase component of pyruvate dehydrogenase complex | Energy generation | CBA | 0.057 | 0.076 | 0.123 |
| Mef1p | Mitochondrial | Mitochondrial translation elongation factor G | Energy generation, protein synthesis | ABC | 0.030 | 0.018 | 0.014 |
| Var1p | Mitochondrial | Mitochondrial small subunit ribosomal protein, mitochondrially coded | Energy generation, protein synthesis | A+ | 0.117 | 0.053 | 0.049 |
| Etf-betap | Mitochondrial | Electron-transferring flavoprotein, beta chain | Energy generation | ABC | 0.162 | 0.095 | 0.044 |
| t-RNA SYNTHETASES | | | | | | | |
| Ygl245wp | Cytoplasmic | Glutamyl-tRNA synthetase | RNA processing, protein synthesis | ABC | 0.054 | 0.030 | 0.002 |
| Dps1p | Cytoplasmic | Aspartyl-tRNA synthetase | Protein synthesis | ABC | 0.299 | 0.214 | 0.148 |
| Ths1p | Cytoplasmic | Threonyl-tRNA synthetase | Protein synthesis | ABC | 0.256 | 0.121 | 0.078 |
| Wrs1p | Cytoplasimc | Tryptophanyl-tRNA synthetase | Protein synthesis | BCA | 0.054 | 0.076 | 0.067 |
| Ydr341cp | Cytoplasmic | Arginine-tRNA synthetase, | Protein synthesis | C− | 0.340 | 0.334 | 0.154 |
| RIBOSOMAL PROTEINS | | | | | | | |
| Rpp0p | Ribosomal | Acidic ribosomal protein A0 | Protein synthesis | ABC | 0.140 | 0.106 | 0.080 |
| Rps3p | Ribosomal | Ribosomal protein S3 | Protein synthesis | ACB | 0.043 | 0.033 | 0.040 |
| Asc1p | Ribosomal | Ribosomal protein of the 40S ribosomal subunit that influences translational efficiency and cell size | Amino acid metabolism, Pol II transcription, protein synthesis | C− | 0.139 | 0.123 | 0.075 |

Copyright © 2001 John Wiley & Sons, Ltd.

Table 2. Continued

| Protein | Cellular localisation | Protein name and biochemical function | Cellular role | Expression group | Average %IOD in CEN.PK2-1B | Average %IOD in FY1679-1D | Average %IOD in W303-1B |
|---|---|---|---|---|---|---|---|
| Rps0Ap | Ribosomal | Ribosomal protein S0, nearly identical to Rps0Bp | Protein synthesis | C− | 0.063 | 0.057 | 0.003 |
| Rps0Bp | Ribosomal | Ribosomal protein S0, nearly identical to Rps0Ap | Protein synthesis | CAB | 0.193 | 0.174 | 0.246 |
| NUCLEOTIDE METABOLISM | | | | | | | |
| Ade12p | Nuclear | Adenylosuccinate synthetase, carries out addition of aspartic acid to IMP with GTP hydrolysis | Nucleotide metabolism | C− | 0.138 | 0.170 | 0.066 |
| Ylr432wp | Unknown | Protein highly similar to Imd2p and IMP dehydrogenase of human and E. coli | Nucleotide metabolism | A− | 0.081 | 0.205 | 0.220 |
| Fcy1p | Unknown | Cytosine deaminase | Nucleotide metabolism | B+ | 0.200 | 0.295 | 0.208 |
| Ynk1p | Unknown | Nucleoside diphosphate kinase, responsible for synthesis of all nucleoside triphosphates except ATP | Nucleotide metabolism | A+ | 0.199 | 0.074 | 0.108 |
| STEROL METABOLISM | | | | | | | |
| Mvd1p | Unknown | Mevalonate diphosphate (MVAPP) decarboxylase (MVA-5-pyrophosphate decarboxylase) | Lipid, fatty-acid, sterol and ergosterol metabolism | A+ | 0.085 | 0.051 | 0.057 |
| CARBOHYDRATES METABOLISM | | | | | | | |
| Tal1p | Cytoplasimc | Transaldolase, component of non-oxidative part of pentose-phosphate pathway | Carbohydrate metabolism | B+ | 0.088 | 0.180 | 0.075 |
| Rhr2p | Unknown | DL-glycerol phosphate phosphatase | Carbohydrate metabolism | A− | 0.076 | 0.155 | 0.145 |
| Hxh2p | Nuclear | Hexokinase II, converts hexoses to hexose phosphates in glycolysis | Carbohydrate metabolism | B+ | 0.050 | 0.077 | 0.043 |
| AMINO ACIDS METABOLISM | | | | | | | |
| Leu1p | Cytoplasimc | 3-Isopropylmalate dehydratase, second step in leucine biosynthesis pathway | Amino acids metabolism | B− | 0.130 | 0.074 | 0.132 |
| Leu1p* | Cytoplasimc | 3-Isopropylmalate dehydratase, second step in leucine biosynthesis pathway | Amino acids metabolism | B− | 0.127 | 0.067 | 0.144 |
| Arg4p | Cytoplasimc | Argininosuccinate lyase, catalyzes the final step in arginine biosynthesis | Amino acids metabolism | ACB | 0.061 | 0.024 | 0.037 |
| Lys7p | Cytoplasimc | Copper chaperone for superoxide dismutase Sod1p | Amino acids metabolism, cell stress | A+ | 0.055 | 0.005 | 0.010 |
| Met17p | Cytoplasmic | O-acetylhomoserine sulfhydrylase (OAH SHLase); converts O-acetylhomoserine into homocysteine | Amino acids metabolism | A+ | 0.367 | 0.225 | 0.247 |

Copyright © 2001 John Wiley & Sons, Ltd.

Table 2. Continued

| Protein | Cellular localisation | Protein name and biochemical function | Cellular role | Expression group | Average %IOD in CEN.PK2-1B | Average %IOD in FY1679-1D | Average %IOD in W303-1B |
|---|---|---|---|---|---|---|---|
| Ilv5p | Mitochondrial | Ketol-acid reductoisomerase, second step in valine and isoleucine biosynthesis pathway | Amino acids metabolism | ACB | 0.111 | 0.076 | 0.100 |
| Hom2p | Unknown | Aspartate-semialdehyde dehydrogenase, second step in common pathway for methionine and threonine biosynthesis | Amino acids metabolism | ACB | 0.111 | 0.076 | 0.100 |
| Aro8p | Unknown | Aromatic amino acid aminotransferase I | Amino acids metabolism | C+ | 0.054 | 0.051 | 0.077 |
| PROTEIN SYNTHESIS, TRANSLOCATION AND MODIFICATION | | | | | | | |
| Cpr3p | Mitochondrial | Cyclophilin (peptidylprolyl cis-trans isomerase or PPlase) of mitochondria | Protein folding | B+ | 0.371 | 0.761 | 0.248 |
| Cpr6p | Cytoplasimc | Cyclophilin (peptidylprolyl cis-trans isomerase or PPlase), interacts with Hsp82p, homolog of mammalian cyclophilin Cyp40 | Protein folding | BCA | 0.035 | 0.089 | 0.065 |
| Hsp82p | Cytoplasimc | Heat-inducible chaperonin homologous to E. coli HtpG and mammalian HSP90 | Protein folding, cell stress | C+ | 0.085 | 0.086 | 0.102 |
| Hsp78p | Mitochondrial | Heat shock protein of the ClpB family of ATP-dependent proteases, mitochondrial | Protein folding, cell stress, protein translocation | A+ | 0.042 | 0.007 | 0.002 |
| Hdp78p | Mitochondrial | Heat shock protein of the ClpB family of ATP-dependent proteases, mitochondrial | Protein folding, cell stress, protein translocation | CAB | 0.046 | 0.037 | 0.050 |
| Ssc1p | Mitochondrial | Mitochondrial protein that acts as an import motor with Tim44p and plays a chaperonin role in | Protein folding, protein translocation | A− | 0.039 | 0.096 | 0.077 |
| Smt3p | Nuclear | Ubiquitin-related protein, becomes conjugated to other proteins in a process requiring ATP, Uba2p, Aos1p, and Ubc9p | Protein modification | B+ | 0.065 | 0.229 | 0.111 |
| Ssb1p | Ribosomal | Heat shock protein of HSP70 family, cytoplasmic | Protein folding, cell stress, protein synthesis | C− | 0.151 | 0.172 | 0.073 |
| Ssb1p* | Nuclear | Heat shock protein of HSP70 family, involved with the translational machinery | Protein folding, cell stress, protein synthesis | CBA | 0.170 | 0.217 | 0.239 |
| Ssb2p | Nuclear | Heat shock protein of HSP70 family, involved with the translational machinery | Protein folding, cell stress, protein synthesis | CBA | 0.051 | 0.075 | 0.086 |
| RIBOFLAVIN BIOSYNTHESIS | | | | | | | |
| Rib3p | Unknown | DBP synthase; (3,4-dihydroxy-2-butanone 4-phosphate synthase), part of the riboflavin biosynthesis pathway | Other metabolism | A− | 0.004 | 0.058 | 0.043 |

Copyright © 2001 John Wiley & Sons, Ltd.

Table 2. Continued

| Protein | Cellular localisation | Protein name and biochemical function | Cellular role | Expression group | Average %IOD in CEN.PK2-1B | Average %IOD in FY1679-1D | Average %IOD in W303-1B |
|---|---|---|---|---|---|---|---|
| Rib4p | Unknown | Riboflavin biosynthesis pathway enzyme, 6,7-dimethyl-8-ribityllumazine synthase | Other metabolism | C+ | 0.206 | 0.066 | 0.478 |
| NITROSATIVE STRESS AND OXIDATIVE STRESS | | | | | | | |
| Yhb1p | Cytoplasimc | Flavohemoglobin involved in protection from nitrosative stress, distantly related to animal hemoglobins | Nitrosative stress | ACB | 0.325 | 0.216 | 0.276 |
| Yhb1p* | Cytoplasimc | Flavohemoglobin involved in protection from nitrosative stress, distantly related to animal hemoglobins | Nitrosative stress | B− | 0.062 | 0.004 | 0.047 |
| Tsa1p | Cytoplasimc | Thioredoxin peroxidase, abundant thiol-specific antioxidant protein that prevents formation of sulfur-containing radicals | Cell stress | B+ | 0.206 | 0.363 | 0.217 |
| Ahp1p | Mitochondrial | Alkyl hydroperoxide reductase,one of five thiol peroxidases | Cell stress | BCA | 0.191 | 0.384 | 0.275 |
| PHOSPHATE METABOLISM | | | | | | | |
| Ipp1p | Cytoplasimc | Inorganic pyrophosphatase, cytoplasmic | Phosphate metabolism | CBA | 0.021 | 0.034 | 0.065 |
| PROTEASOME COMPLEX | | | | | | | |
| Rpn12p | Nuclear | Non-ATPase component of 26S proteasome complex; required for activation of Cdc28p protein kinase | Protein degradation | C− | 0.063 | 0.057 | 0.003 |
| Rpt4p | Nuclear | Component of 26S proteasome complex and member of the AAA family of ATPases | Protein degradation | A+ | 0.056 | 0.035 | 0.036 |
| UNKNOWN FUNCTION | | | | | | | |
| Ydl124wp | Unknown | Protein of unknown function | Unknown | B+ | 0.017 | 0.040 | 0.021 |
| Yhr029cp | Unknown | Protein of unknown function | Unknown | C+ | 0.051 | 0.054 | 0.079 |
| Yor285wp | Unknown | Protein with similarity to Drosophila melanogaster heat shock protein 67B2 | Unknown | A+ | 0.325 | 0.136 | 0.167 |
| Ypl004cp | Unknown | Protein with weak similarity to tropomyosin | Unknown | B+ | 0.061 | 0.098 | 0.063 |

Copyright © 2001 John Wiley & Sons, Ltd.

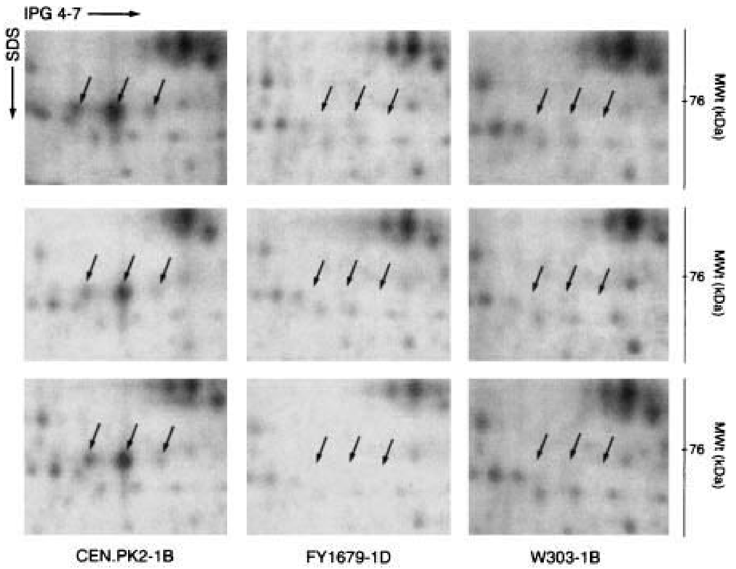

**Figure 3.** A close up on a region of the 2DGE pattern from CEN.PKC-1B, FY1679-1D and W303-1B strains (in triplicate) showing 3 protein spots that have been identified as major coat protein Gagp from *Saccharomyces cerevisiae* virus ScV-L-A. The three isoforms of the protein indicate a possible post-translational modification (e.g. N-terminal acetylatation)

confirm the N-acetylation of the Gagp protein by mass spectrometry.

### Sterol metabolism

Mvd1p was expressed at a higher level in the CEN.PK2-1B strain. This enzyme decarboxylates mevalonate diphosphate and produces isopentenyl diphosphate (IPP) that is used for synthesis of sterols and for protein farnesylation or geranylgeranylation. Interestingly, this protein is less abundant in FY1679-1D and W303-1B compared to CEN.PK2-1B by about 63%. Differences in lipid metabolism between these strains have been reported before (Daum *et al.*, 1999) where it has been shown that for example the FY1679 strain has lower levels of triacylglycerols and sterol compared to the other two strains.

### Expression of t-RNA synthetases

Five aminoacyl-tRNA synthetases have been identified in this study: Tryptophanyl-tRNA synthetase (Wrs1p), Glutamyl-tRNA synthetase (Ygl245wp), Arginine-tRNA synthetase, (Ydr341cp), Aspartyl-tRNA synthetase (Dps1p) and Threonyl-tRNA synthetase (Ths1p) (Table 2).

Three of the enzymes (Ygl245wp, Dps1p and Ths1p) belong to the ABC expression group (Table 2) and are expressed at a high level in CEN.PK2-1B. Ydr341cp was classified in the C- group, its expression is down-regulated in W303-1B and it is highly expressed in CEN.PK2-1B. Only Tryptophanyl-tRNA synthetase was classified into the BCA group, and it is more abundant in FY1679-1D and in W303-1B than in the CEN.PK2-1B strain.

The family of aminoacyl-tRNA synthetases play a key role in the readout of the genetic code catalysing the attachment of a given amino acid to the corresponding tRNA and indirectly take part in protein synthesis. The elevated expression of four aminoacyl-tRNA synthetases in CEN.PK2-1B strain could indicate a more intensive protein synthesis. This is, however, not confirmed as all the strains exhibited the same growth rate (about 90 min per generation) in logarithmic growth phase conditions used here.

### Ribosomal proteins Rps0

Rps0Ap and Rps0Bp are two nearly identical ribosomal proteins (95% identity) (Demianova *et al.*, 1996) that are required for assembly and stability of the 40S ribosomal subunit.

Demianova and co-workers have observed that disruption of the *RPS0A* gene in W303 results in a slow growth phenotype, and that disruption of the *RPS0B* gene results in an even slower growth rate and that the steady state levels of *RPS0B* mRNA are

Copyright © 2001 John Wiley & Sons, Ltd. 

higher than the *RPS0A* mRNA. Based on the ability of plasmid-borne copies of *RPS0A* and *RPS0B* genes to complement the growth defects associated with disruptions in either gene they have postulated that these two proteins are functionally equivalent but Rps0Bp makes a greater contribution to the pool of Rps0 molecules (Demianova *et al.*, 1996).

Comparing the relative expression of Rps0Bp and Rps0Ap in each strain we have observed that the expression ratio between Rps0Bp and Rps0Ap in the CEN.PK2-1B and FY1679-1D is 3.08 and 3.05 whereas in the W303-1B strain it is 81.6. In this study we have observed that the expression level of the Rps0Ap in W303-1B is significantly lower than in the CEN.PK2-1B and FY1679-1D (the protein spot was less intense by a factor of 21 and 19 respectively) (compare Figure 4 and Table 2). On the contrary, the expression of the Rps0Bp was higher in W303-1B than in the other two strains by factor of 1.2 (CEN.PK2-1B) and 1.4 (FY1679-1D). Thus our results confirm the finding of Demianova and co-workers and additionally indicate that the difference in the differences in the relative amounts of Rps0Ap and Rps0Bp is most pronounced in the W303 strain.

### Possible post-translational modifications detected by 2DGE

Five of the proteins selected and identified in this study (Leu1p, Hsp78p, Ssb1p, Dsk2p and Yhb1p) were found in two or three spots. All these proteins have been identified as two spots placed in a row very close to each other (Figure 2A, spots marked with star (*)). This kind of spot pattern indicates a post-translational modification that changes the pI of the protein, without major changes of its molecular mass (change of mass up to 1% will not be distinguishable on a 2DGE of this kind).

Leu1p, 3-Isopropylmalate dehydratase, is involved in the second step of the leucine biosynthesis pathway and no protein modification site has been identified or predicted. Both protein spots are significantly less expressed in the FY1679-1D indicating that the observed difference results from overall decreased expression of Leu1p and not only of one of its isoforms.

Hsp78p has been identified in two spots in a row, separated by a smaller spot containing a C-terminal truncated form of Met6p. The more neutral spot is significantly more expressed in CEN.PK2-1B and the more acidic protein was most abundant in W303-1B, moderately in CEN.PK2-1B and least in FY1679-1D (Figure 2A and Table 2). Based on the protein spot position and the mass spectrometry peptide coverage, we can deduce that the N-terminal putative mitochondrial leader sequence has been cleaved off. Ssb1p is a heat shock protein of HSP70 family, involved with the translational machinery. The two spots show similar expression patterns (Figure 2A, Table 2). It is most abundantly expressed in the W303-1B strain, less in the FY1679-1D strain and at the lowest level in the CEN.PK2-1B strain. Ssb1p has been shown to be N-acetylated by the Nat1p-Ard1p N-terminal acetyltransferase (Polevoda *et al.*, 1999), which could account for the observed shift in the position.

Dsk2p is a protein that is required, with Rad23p, for duplication of the spindle pole body. This protein was found to be up-regulated in the more acidic spot and down regulated in the more neutral spot in CEN.PK2-1B (Table 2). This would indicate a post-translational modification seen in CEN.PK2-1B, that is less pronounced in the FY1679-1D and

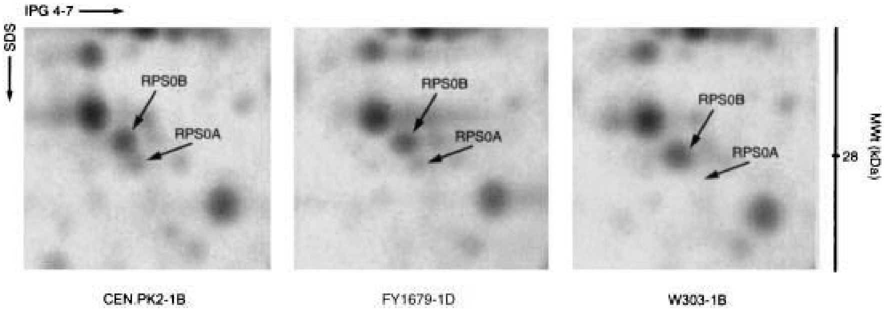

**Figure 4.** A close up on a region of the 2DGE pattern from CEN.PKC-1B, FY1679-1D and W303-1B strains showing protein spots that have been identified as Rps0Ap and Rps0Bp, two nearly identical ribosomal proteins. Note that the spot containing Rps0Ap in W303-1B is almost invisible, indicating a very low expression level

Copyright © 2001 John Wiley & Sons, Ltd. 

W303-1B strains. Sequence-based methods predict possible N-terminal acetylation (Huang *et al.*, 1987) and a mass peak of 824.43 Da that corresponds to the N-terminal sequence (SLNIHIK) of Dsk2p (after trypsin digestion with the N-terminal methionine cleaved off) was detected in the more neutral spot. In the spectrum obtained from the more acidic spot we have found a mass peak of 866.42 Da that corresponds to the same N-terminal peptide of Dsk2p (after trypsin digestion). The increased mass of the peak by 42.01 Da confirms the prediction that the sequence is modified by N-terminal acetylation but the results demonstrate that both the modified and unmodified proteins exist.

Yeast flavohemoglobin (Yhb1p) is related to globins and a reductase family (Zhu *et al.*, 1992) and is involved in protecting the cell from nitrosative stress (Liu *et al.*, 2000). (Figure 2 and Table 2). Both spots identified here, especially the less abundant and more acidic spot, showed the lowest expression level in the FY1679-1D strain. It has been reported that the N-terminus of the Yhb1p protein is unmodified (Zhu *et al.*, 1992). Unfortunately the N-terminus was not detected in these studies and none of the other detected peptides gave any indication as to what type of post-translational modification could be related to the observed shift in the spot position.

### Conclusions

This study represents the first comparison of protein expression levels between the haploid strains derived from yeast wild type CEN.PK2, FY1679 and W303 strains. It reports differences between the strains shown by 73 protein spots different between CEN.PK2-1B and FY1679-1D, 67 between CEN.PK2-1B and W303-1B and 39 spots different between FY1679-1D and W303-1B. These data show that the FY1679-1D and W303-1B strains are more similar to each other than to the CEN.PK2-1B strain, in agreement with their genealogy (FY1679 and W303 have a common ancestor strain S288C). Undoubtedly the observed differences in protein expression and post-translational modification influence the molecular and biochemical characteristics of the cells and possibly result in different phenotypes of yeast mutants observed in these strains. Therefore it is very important to identify and understand these differences prior to functional interpretation of phenotypic characteristics of yeast mutants obtained in functional analysis studies. This study identifies 62 proteins that are changed between the strains and provides a valuable source of data for the interpretation of differences in yeast mutant phenotypes observed in CEN.PK2, FY1679 and W303 derived strains.

### Acknowledgements

This work was supported by the European Commission grant (No. BIO4CT972361) for the PAGES project in Centre for Proteome Analysis in Life Sciences, University of Southern Denmark Odense University, International Science Park Odense. This work is also a part of the activities of the Centre for Experimental Bioinformatics sponsored by Danish National Research Foundation. We would like to thank to Arek Nawrocki for inspiring discussions, Andrea Maria Lorentzen, Lene Storgaard and Chris Christensen for excellent technical support, Jef Faridi for computer service and database maintenance and Anders M. Jensen for expert image analysis. We would like to express our special gratitude to Rodney Rothstein who provided invaluable information about genealogy of W303 strain.

### References

Bilsland E, Dahlen M, Sunnerhagen P. 1998. Genomic disruption of six budding yeast genes gives one drastic example of phenotype strain-dependence. *Yeast* **14**: 655–664.

Bonhivers M, Carbrey JM, Gould SJ, Agre P. 1998. Aquaporins in *Saccharomyces*. Genetic and functional distinctions between laboratory and wild-type strains. *J Biol Chem* **273**: 27565–27572.

Boucherie H, Sagliocco F, Joubert R, Maillet I, Labarre J, Perrot M. 1996. Two-dimensional gel protein database of *Saccharomyces cerevisiae*. *Electrophoresis* **17**: 1683–1699.

Bradford MM. 1976. A rapid and sensitive method for the quantitation of microgram quantities of protein utilizing the principle of protein-dye binding. *Anal Biochem* **72**: 248–254.

Cairns BR, Erdjument-Bromage H, Tempst P, Winston F, Kornberg RD. 1998. Two actin-related proteins are shared functional components of the chromatin-remodeling complexes RSC and SWI/SNF. *Mol Cell* **2**: 639–651.

Costanzo MC, Crawford ME, Hirschman JE, *et al.* 2001. YPD, PombePD and WormPD: model organism volumes of the BioKnowledge library, an integrated resource for protein information. *Nucleic Acids Res* **29**: 75–79.

Daum G, Tuller G, Nemec T, *et al.* 1999. Systematic analysis of yeast strains with possible defects in lipid metabolism. *Yeast* **15**: 601–614.

Demianova M, Formosa TG, Ellis SR. 1996. Yeast proteins related to the p40/laminin receptor precursor are essential components of the 40 S ribosomal subunit. *J Biol Chem* **271**: 11383–11391.

Duenas E, Vazquez de Aldana CR, de Cos T, Castro C, Henar Valdivieso M. 1999. Generation of null alleles for the functional analysis of six genes from the right arm of *Saccharomyces cerevisiae* chromosome II. *Yeast* **15**: 615–623.

Copyright © 2001 John Wiley & Sons, Ltd.

Elion EA, Brill JA, Fink GR. 1991. FUS3 represses CLN1 and CLN2 and in concert with KSS1 promotes signal transduction. *Proc Natl Acad Sci U S A* **88**: 9392–9396.

Entian K-D, Kotter P. 1998. Yeast mutant and plasmid collections. *Methods in Microbiology* **26**: 431–449.

Entian K-D, Schuster T, Hegemann JH, *et al.* 1999. Functional analysis of 150 deletion mutants in *Saccharomyces cerevisiae* by a systematic approach. *Mol Gen Genet* **262**: 683–702.

Fey SJ, Nawrocki A, Larsen MR, *et al.* 1997. Proteome analysis of *Saccharomyces cerevisiae*: a methodological outline. *Electrophoresis* **18**: 1361–1372.

Gaisne M, Becam AM, Verdiere J, Herbert CJ. 1999. A 'natural' mutation in *Saccharomyces cerevisiae* strains derived from S288c affects the complex regulatory gene HAP1 (CYP1). *Curr Genet* **36**: 195–200.

Goto K, Fukuda H, Kichise K, Kitano K, Ilara S. 1991. Cloning and nuclcotide sequence of the KHS killer gene of *Saccharomyces cerevisiae*. *Agric Biol Chem* **55**: 1953–1958.

Grava S, Dumoulin P, Madania A, Tarassov I, Winsor B. 2000. Functional analysis of six genes from chromosomes XIV and XV of *Saccharomyces cerevisiae reveals* YOR145c as an essential gene and YNL059c/ARP5 as a strain-dependent essential gene encoding nuclear proteins. *Yeast* **16**: 1025–1033.

Hajji K, Clotet J, Arino J. 1999. Disruption and phenotypic analysis of seven ORFs from the left arm of chromosome XV of *Saccharomyces cerevisiae*. *Yeast* **15**: 435–441.

Hoogland C, Sanchez JC, Tonella L, *et al.* 2000. The 1999 SWISS-2DPAGE database update. *Nucleic Acids Res* **28**: 286–288.

Huang S, Elliott RC, Liu PS, *et al.* 1987. Specificity of cotranslational amino-terminal processing of proteins in yeast. *Biochemistry* **26**: 8242–8246.

Jensen ON, Larsen MR, Roepstorff P. 1998. Mass spectrometric identification and microcharacterization of proteins from electrophoretic gels: strategies and applications. *Proteins* (Suppl. 2): 74–89.

Joubert R, Brignon P, Lehmann C, Monribot C, Gendre F, Boucherie H. 2000. Two-dimensional gel analysis of the proteome of lager brewing yeasts. *Yeast* **16**: 511–522.

Kron SJ. 1997. Filamentous growth in budding yeast. *Trends Microbiol* **5**: 450–454.

Kucharczyk R, Gromadka R, Migdalski A, Slonimski PP, Rytka J. 1999. Disruption of six novel yeast genes located on chromosome II reveals one gene essential for vegetative growth and two required for sporulation and conferring hypersensitivity to various chemicals. *Yeast* **15**: 987–1000.

Lieberman PM, Schmidt MC, Kao CC, Berk AJ. 1991. Two distinct domains in the yeast transcription factor IID and evidence for a TATA box-induced conformational change. *Mol Cell Biol* **11**: 63–74.

Liu H, Styles CA, Fink GR. 1996. *Saccharomyces cerevisiae* S288C has a mutation in FLO8, a gene required for filamentous growth. *Genetics* **144**: 967–978.

Liu LM, Zeng M, Hausladen A, Heitman J, Stamler JS. 2000. Protection from nitrosative stress by yeast flavohemoglobin. *Proc Nat Acad Sci U S A* **97**: 4672–4676.

Lopez MC, Sanchez M, Ferminan E, Dominguez A. 1998. Disruption of six *Saccharomyces cerevisiae* genes from chromosome IV and basic phenotypic analysis of deletion mutants. *Yeast* **14**: 1199–1208.

Mortimer RK, Johnston JR. 1986. Genealogy of principal strains of the yeast genetic stock center. *Genetics* **113**: 35–43.

Nawrocki A, Larsen MR, Podtelejnikov AV, *et al.* 1998. Correlation of acidic and basic carrier ampholyte and immobilized pH gradient two-dimensional gel electrophoresis patterns based on mass spectrometric protein identification. *Electrophoresis* **19**: 1024–1035.

Norbeck J, Blomberg A. 1997. Two-dimensional electrophoretic separation of yeast proteins using a non-linear wide range (pH 3–10) immobilized pH gradient in the first dimension; reproducibility and evidence for isoelectric focusing of alkaline (pI > 7) proteins. *Yeast* **13**: 1519–1534.

Perrot M, Sagliocco F, Mini T, *et al.* 1999. Two-dimensional gel protein database of *Saccharomyces cerevisiae* (update 1999). *Electrophoresis* **20**: 2280–2298.

Polevoda B, Norbeck J, Takakura H, Blomberg A, Sherman F. 1999. Identification and specificities of N-terminal acetyltransferases from *Saccharomyces cerevisiae*. *Embo Journal* **18**: 6155–6168.

Prior C, Potier S, Souciet JL, Sychrova H. 1996. Characterization of the NHA1 gene encoding a Na+/H+-antiporter of the yeast *Saccharomyces cerevisiae*. *FEBS Lett* **387**: 89–93.

Rohde JR, Trinh J, Sadowski I. 2000. Multiple signals regulate GAL transcription in yeast. *Mol Cell Biol* **20**: 3880–3886.

Rothstein RJ, Esposito RE, Esposito MS. 1977. The effect of ochre suppression on meiosis and ascospore formation in *Saccharomyces*. *Genetics* **85**: 35–54.

Rothstein RJ, Sherman F. 1980a. Dependence on mating type for the overproduction of iso-2-cytochrome c in the yeast mutant CYC7-H2. *Genetics* **94**: 891–898.

Rothstein RJ, Sherman F. 1980b. Genes affecting the expression of cytochrome c in yeast: genetic mapping and genetic interactions. *Genetics* **94**: 871–889.

Rothstein RJ. 1977. A genetic fine structure analysis of the suppressor 3 locus in *Saccharomyces*. *Genetics* **85**: 55–64.

Rothstein RJ. 1983. One-step gene disruption in yeast. *Methods Enzymol* **101**: 202–211.

Shevchenko A, Jensen ON, Podtelejnikov AV, *et al.* 1996. Mann M. Linking genome and proteome by mass spectrometry: large-scale identification of yeast proteins from two dimensional gels. *Proc Natl Acad Sci U S A* **93**: 14440–14445.

Tercero JC, Dinman JD, Wickner RB. 1993. Yeast MAK3 N-acetyltransferase recognizes the N-terminal four amino acids of the major coat protein (gag) of the L-A double-stranded RNA virus. *J Bacteriol* **10**: 3192–3194.

Thierry A, Fairhead C, Dujon B. 1990. The complete sequence of the 8.2 kb segment left of MAT on chromosome III reveals five ORFs, including a gene for a yeast ribokinase. *Yeast* **6**: 521–534.

Thomas BJ, Rothstein R. 1989. Elevated recombination rates in transcriptionally active DNA. *Cell* **56**: 619–630.

Wallis JW, Chrebet G, Brodsky G, Rolfe M, Rothstein R. 1989. A hyper-recombination mutation in *S. cerevisiae* identifies a novel eukaryotic topoisomerase. *Cell* **58**: 409–419.

Winston F, Dollard C, Ricupero-Hovasse SL. 1995. Construction of a set of convenient *Saccharomyces cerevisiae* strains that are isogenic to S288C. *Yeast* **11**: 53–55.

Yu Y, Eriksson P, Stillman DJ. 2000. Architectural transcription factors and the SAGA complex function in parallel pathways to activate transcription. *Mol Cell Biol* **20**: 2350–2357.

Zhu H, Riggs AF. 1992. Yeast flavohemoglobin is an ancient protein related to globins and a reductase family. *Proc Natl Acad Sci U S A* **89**: 5015–5019.

Copyright © 2001 John Wiley & Sons, Ltd. 
